# A patient with neurofibromatosis type 1 presenting with bilateral frontal lobe infarctions following anterior communicating artery aneurysm rupture

**DOI:** 10.1177/2050313X19841151

**Published:** 2019-04-04

**Authors:** HMMTB Herath, Nilukshana Yogendranathan, Aruna Kulatunga

**Affiliations:** The National Hospital of Sri Lanka, Colombo, Sri Lanka

**Keywords:** Neurofibromatosis type 1, von Recklinghausen’s disease, anterior communicating artery aneurysm rupture, bilateral frontal lobe infarctions

## Abstract

Neurofibromatosis is a neurocutaneous genetic condition with dysplasia of the mesodermal and ectodermal tissues. Vascular abnormalities are well recognized in neurofibromatosis and cerebral aneurysms are rarely reported in literature. Here, we present a 20-year-old Sri Lankan female presented with headache, altered personality, disinhibited behaviour, and urinary incontinence. On imaging, she was found to have infarctions of both frontal lobes and evidence of a ruptured anterior communicating artery aneurysm with a small subarachnoid haemorrhage. Another small middle cerebral artery aneurysm was also seen in the angiogram. She was managed conservatively and gradually recovered. Because aneurysms in neurofibromatosis are usually asymptomatic and as rupture of such an aneurysm is rare, regular vascular screening is not recommended to all patients with neurofibromatosis. This is the first case report in literature in which a patient with neurofibromatosis presented with infarctions of both frontal lobes due to rupture of an anterior communicating artery aneurysm.

## Background

Neurofibromatosis (NF) is a neurocutaneous genetic condition with dysplasia of the mesodermal and ectodermal tissues. Vascular abnormalities are well recognized in NF-1, and the renal arteries are the most frequently involved. Abnormalities in the cerebral vasculature such as moyamoya syndrome, intracranial aneurysms, narrowed or ectatic vessels, and vascular stenosis and strokes in infants are also documented in several case reports.^1–5^ Only a limited number of cases of intracranial aneurysms are reported in patients with NF-1. Some studies have identified neurofibromin as a novel regulator of Ras activity in vascular smooth muscular cells and provide a framework for understanding cardiovascular disease in NF-1 patients.^6^ Li et al.^7^ demonstrated genetic and pharmacological evidence that NF-1 (+/−) myeloid cells are the cellular triggers for aneurysm formation in NF-1 vasculopathy. Here, we present a young female with NF-1 who presented with infarctions of both frontal lobes whose cerebral angiogram revealed the possible rupture of an anterior communicating artery (AComA) aneurysm and a small middle cerebral artery (MCA) aneurysm.

## Case presentation

A 20-year-old female, with NF type 1, presented with sudden onset of headache, vomiting, and altered behaviour for 4 days. She had begun to act in a disinhibited manner and was using offensive language towards her family members. She also had urinary incontinence. Headache was severe and continuous.

On examination, the patient had multiple neurofibromata, café au lait spots and Leish nodules of the iris. There was no family history of NF. There were no features of meningism or any focal neurological signs. We were unable to assess her memory, higher functions and frontal lobe functions properly on admission due to her behaviour.

**Figure 1.**
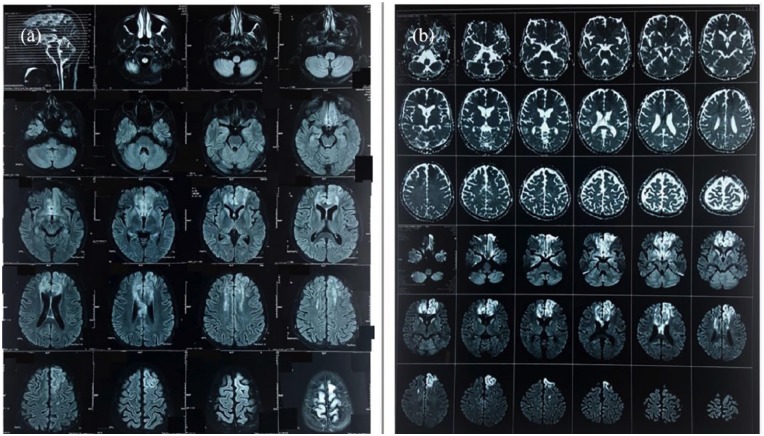
(a) MRI FLAIR axial images showing cortical and subcortical white matter signal changes in both frontal lobes compatible with bilateral frontal lobe acute infarctions in the anterior cerebral artery territory. (b) Restriction in DWI in both frontal lobes compatible with bilateral frontal lobe acute infarctions.

**Figure 2.**
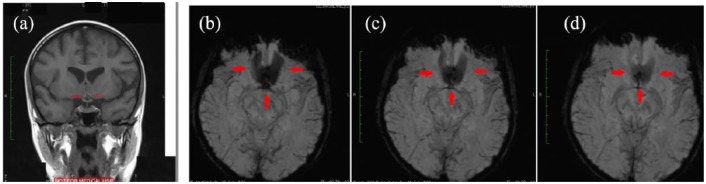
(a) MRI T1 coronal images showing subacute heamorrhage (red arrows) in the anterior communicating artery area. (b–d) SWI showing loss of signal in the anterior communicating artery area (red arrows) suggestive of haemorrhage.

**Figure 3.**
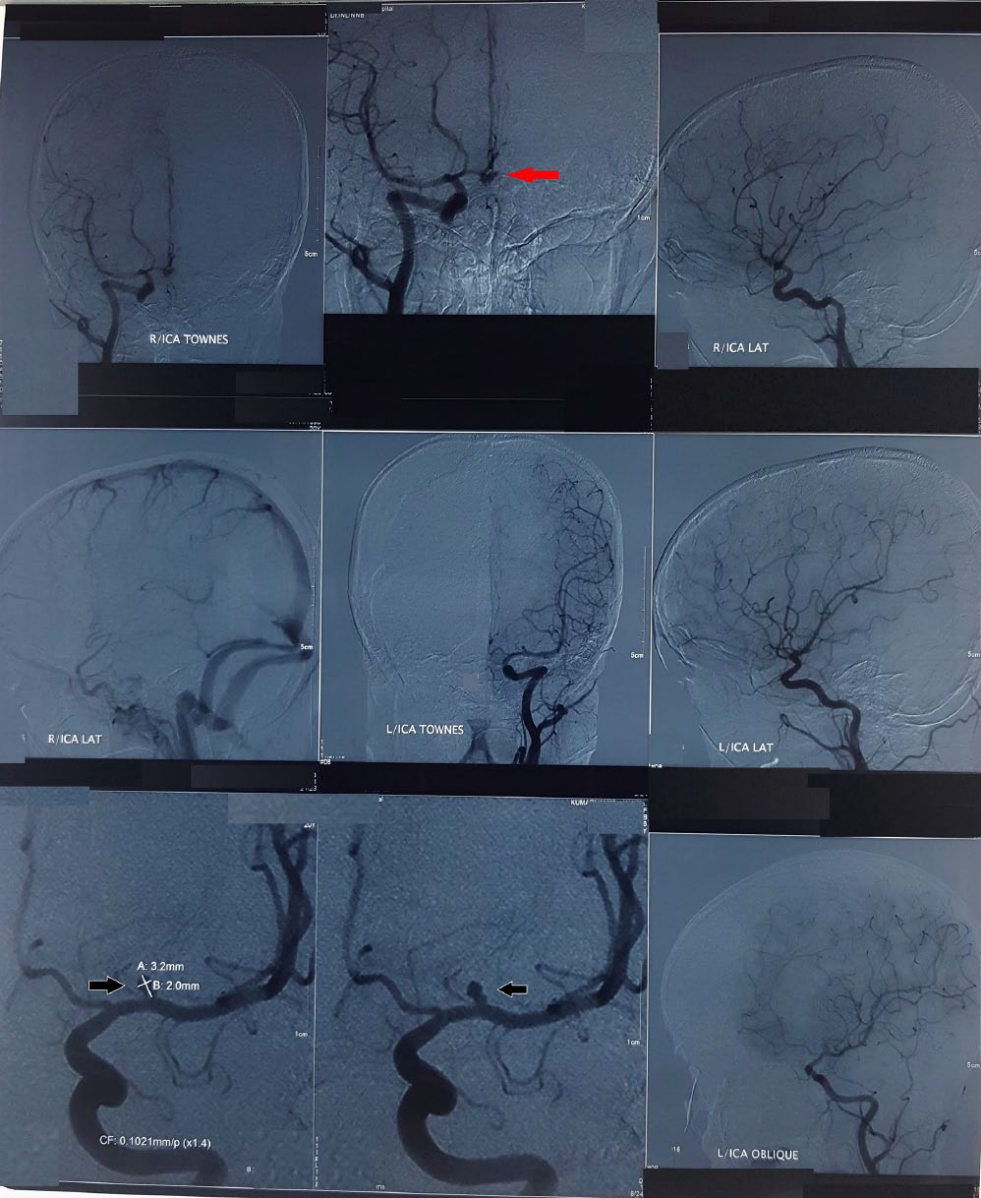
DSA images: red arrow shows possible ruptured anterior communicating artery aneurysm. Black arrows show 3.2 mm × 2 mm left MCA aneurysm.

Full blood count, liver function tests, renal function tests, thyroid function tests and inflammatory markers were normal. Noncontrast computed tomography (NCCT) scan of the brain, done on admission (4 days after the onset of headache), revealed hypodense areas in both frontal lobes. Subsequent cerebrospinal fluid (CSF) analysis was normal with the absence of cells and normal protein and sugar levels. Magnetic resonance imaging (MRI) with magnetic resonance venogram/magnetic resonance angiogram (MRV/MRA) (10 days after the onset of headache) concluded bilateral frontal lobe infarcts (Figure 1(a)) with restriction in diffusion-weighted imaging (DWI) (Figure 1(b)) along with possible narrowing at the origins of anterior cerebral arteries (ACA) and suggesting spasms of bilateral ACA. A recent bleed at the anterior communicating artery (AComA) was also evident in MRI (Figure 2(a)) and susceptibility weighted imaging (SWI) (Figure 2(b)). Small aneurysm was also seen at the left MCA. She was then subjected to digital subtraction angiogram (DSA) (24 days after the onset of headache), which revealed a possible ruptured aneurysm of AComA (Figure 3). Furthermore, a left-sided MCA saccular aneurysm (3.2 mm × 2 mm) was also detected (Figure 3). Neurosurgical opinion was to manage conservatively because of lack of facilities for intervention. The two-dimensional (2D) echo and the rest of the aortic and renal angiograms were normal.

Her altered personality persisted for 4 weeks and then gradually improved to normal. Her memory was intact and speech, motor functions and urinary continence were normal after 4 weeks.

## Discussion and conclusion

Our patient was diagnosed with NF-1 using National Institutes of Health criteria.^8^ Cerebrovascular abnormalities are well documented in patients with NF-1, and since our patient did not have any other risk factors for development of cerebral aneurysms at a younger age,^9^ we hypothesized a possible link between NF and cerebral aneurysm in this case.

The patient we describe here presented with headache and vomiting for 4 days, and the CT scan of the brain was suggestive of bilateral frontal lobe infarctions. Initially, we thought of vasculitis, reversible cerebral vasoconstriction syndrome and drug-induced cerebral infarcts. However, intravenous antibiotics were also added suspecting encephalitis. CT scan did not have evidence of subarachnoid haemorrhage (SAH) and the CSF analysis done on day 5 of the illness did not reveal any abnormality. CSF for xanthochromia was not analysed because there was no suspicion of SAH at that time. Because of the limited facilities, MRI was done on day 10 of the illness and confirmed bilateral frontal lobe infarctions. MRA showed bilateral ACA spasms. Severe sudden onset headache, bilateral frontal lobe infarctions and ACA spasms were suggestive of possible AComA aneurysm rupture. Therefore, we had a careful second look at the MRI that revealed a recent bleed in the area of AComA. SWI confirmed this recent bleed. With the suspicion of AComA aneurysm, DSA was done and that confirmed AComA aneurysm. This time both the ACA were visualized without spasms. With the DSA evidence of aneurysm and MRI evidence of recent bleeding, we concluded that our patient had a rupture of an AComA aneurysm with a small SAH. Because of this, both cerebral arteries went into spasm leading to bilateral frontal lobe infarctions. The origin of ACA was not clearly seen on initial MRA, but seen later in DSA done several days later. This could have been due to the initial spasm, which also limited the size of the SAH making it too small to be visualized in the CT scan. More sensitive imaging techniques (MRI with SWI sequence) were required to detect the bleed. A timely CT angiogram would have also helped us in diagnosing the aneurysms early.

We did not have facilities for radiological interventions at that time. According to International Study of Unruptured Intracranial Aneurysms (ISUIA), the rates of aneurysmal rupture were lower in smaller aneurysms, and the optimum size cut-off point in this study for defining low risk of rupture was 7 mm.^10^ The MCA aneurysm was small in our patient, and therefore, we planned to manage her conservatively but clippings’ surgery to both aneurysms was an option.

Patients with SAH due to ruptured aneurysms of the AComA have been described in literature as having a classical triad of symptoms (memory loss, confabulation and altered personality) known as ‘anterior communicating artery syndrome’.^11–13^ Our patient also had altered personality, impulsive behaviour, urinary incontinence and impaired memory, which is suggestive of frontal lobe pathology.

Usually, aneurysms in NF-1 are asymptomatic and rupture of an aneurysm is rare. Because clinically significant lesions are relatively uncommon, the role of routine vascular screening in patients with NF-1 has not been evaluated in trials and regular vascular screening is not recommended to all NF-1 patients.^12^ On clinical suspicion, selective imaging is advised, and the follow-up studies should be the same as for patients without NF-1.^14^

## Conclusion

In conclusion, in this study, we describe a young female with NF who presented with bilateral frontal lobe infarctions following AComA aneurysm rupture. Only limited cases of intracranial aneurysms are reported in patients with NF-1. This case report highlights the importance of investigating intracranial aneurysm rupture in patients with NF who present with cerebral infarcts especially in the absence of other vascular risk factors.
